# Machine learning-based nomogram for 30-day mortality prediction for patients with unresectable malignant biliary obstruction after ERCP with metal stent: a retrospective observational cohort study

**DOI:** 10.1186/s12893-023-02158-5

**Published:** 2023-08-30

**Authors:** Zongdong Zhu, Kaixin Hu, Fengqing Zhao, Wen Liu, Hongkun Zhou, Zongliang Zhu, Huangbao Li

**Affiliations:** 1 Jiaxing University Master Degree Cultivation Base, Zhejiang Chinese Medical University, Jiaxing, Zhejiang China; 2 Department of Hepatobiliary and Pancreatic Surgery, First Hospital of Jiaxing, Affiliated Hospital of Jiaxing University, Jiaxing, Zhejiang China; 3 Henan University of Science and Technology, Luoyang, Henan China

**Keywords:** Endoscopic retrograde cholangiopancreatographies, Nomogram, Stent, Malignant bile duct obstruction, Biliary drainage, Mortality

## Abstract

**Background:**

This study aimed to investigate the risk factors for 30-day mortality in patients with malignant biliary obstruction (MBO) after endoscopic retrograde cholangiopancreatography (ERCP) with endobiliary metal stent placement. Furthermore, we aimed to construct and visualize a prediction model based on LASSO-logistic regression.

**Methods:**

Data were collected from 245 patients who underwent their first ERCP with endobiliary metal stent placement for unresectable MBO between June 1, 2013, and August 31, 2021. Univariable and multivariable logistic regression analyses were conducted to identify the risk factors for 30-day mortality. We subsequently developed a logistic regression model that incorporated multiple parameters identified by LASSO regression. The model was visualized and the nomogram was plotted. Risk stratification was performed based on nomogram-derived scores.

**Results:**

The 30-day mortality rate was 10.7% (23/245 patients). Distant metastasis, total bilirubin, post-ERCP complications, and successful drainage were independent risk factors of 30-day mortality. The variables screened by LASSO regression, including distant metastasis, total bilirubin, post-ERCP complications, and successful drainage, were incorporated into the logistic model. The results were visualized through a nomogram based on the model. To assess the model’s performance, discrimination was evaluated using the area-under-the-curve values obtained from receiver operating characteristic analyses with 10-fold cross-validation in the training group and validated in the testing group. The calibration curve showed the good predictive ability of the model. Decision curve analysis is used to evaluate the clinical application of nomogram. Finally, we performed risk stratification based on the risk calculated using the nomogram. Patients were assigned to the low-, moderate-, and high-risk groups based on their probability scores. The Kaplan–Meier survival curves for the different nomogram-based groups were significantly different (*p* < 0.001).

**Conclusions:**

We developed a nomogram using the LASSO-logistic regression model to forecast the 30-day mortality rate in patients who had undergone ERCP with endobiliary metal stent placement due to MBO. This nomogram can assist in identifying individuals at high-risk of 30-day mortality following ERCP.

## Background

Malignant biliary obstruction (MBO) is caused by the direct invasion or compression of the bile duct by pancreatic cancer, cholangiocarcinoma, gallbladder cancer, hepatocellular carcinoma, ampullary cancer, and metastasis from other primary lesions [1]. Surgical resection is the only curative treatment. However, due to the lack of obvious pre-disease symptoms, patients with MBO usually present at advanced and unresectable stages at diagnosis [2, 3]. These patients often have jaundice, itching, discomfort, anorexia, and weight loss, which seriously affect their quality of life [2, 4–6]. Additionally, chemotherapy initiation is frequently postponed to minimize the risk of hepatotoxicity caused by biliary stasis-induced liver function impairment [7–10]. Furthermore, patients diagnosed with unresectable MBO frequently have a poor prognosis, with a five-year survival rate typically below 5% [2, 5, 11].

Endoscopic retrograde cholangiopancreatography (ERCP)-guided endobiliary drainage with stent placement is the primary therapeutic approach to alleviate symptoms and facilitate chemotherapy for patients with unresectable MBO. This treatment has low invasiveness, high efficacy, high patient acceptance, and relatively low complication and mortality rates [10]. In addition, restoring bile circulation conforms to human physiology, thereby avoiding electrolyte disturbances caused by extracorporeal bile drainage [10, 12]. Its survival rates are better when compared with percutaneous transhepatic bile duct drainage and surgical drainage [13–15].

Nevertheless, ten percent of patients with unresectable MBO still have poor outcomes after stent placement, with some patients having elevated total bilirubin (TB) levels or dying within 30 days after surgery [16]. Therefore, it is important to assess the risk factors influencing 30-day mortality. However, few studies have been conducted in this area. Bilirubin sludge damages hepatocytes and affects the metabolic, immune, and coagulation functions of the liver, which may contribute to poor patient outcomes. Several clinical studies have investigated the predictors of mortality following endoscopic endobiliary stent placement, but these studies have utilized univariable and multivariable analyses, which may have limitations in handling multicollinearity between variables [17–19]. Machine learning (ML) is a scientific discipline concerning how computers learn from data [20]. The use of ML has significant value in clinical research because it helps us to accurately search for risk factors leading to target outcome events from a multitude of clinical parameters, eliminating manual aspects of data omission, multicollinearity, and statistical over-fitting problems [21].

The primary objective of this study was to investigate the clinical predictors of 30-day mortality in patients who underwent ERCP with endobiliary metal stent placement. A 30-day mortality prediction model was developed and internally validated. This study further aimed to help clinicians distinguish patients with high-risk factors and to improve palliative care selection by developing predictive models.

## Methods

### Patient selection

This single-center, retrospective, observational cohort study was conducted at the First Hospital of Jiaxing. Eligible patients underwent their first ERCP with endobiliary metal stent placement for unresectable MBO between June 1, 2013, and August 31, 2021. Eighty percent of the data were randomly classified as the training cohort and the remaining 20% were used as the test cohort. The analyzed variables included sex, age, obstruction site, cholangitis before stent insertion, post-ERCP complications, Child–Pugh classification, ascites, white blood cell count, red blood cell count, type of malignancy, hemoglobin, platelet, prothrombin time (PT), TB, albumin, alanine aminotransferase (ALT), aspartate aminotransferase (AST), creatinine, distant metastasis, lymph node metastasis, stent placement, successful drainage, and survival in days. The follow-up ended in December 2021, and the primary endpoint was 30-day mortality, which was calculated as the interval between the date of surgery and the date of death.

The inclusion criteria were as follows:

- patients diagnosed with malignant tumors by pathological or imaging examination that did not undergo surgery owing to tumor invasion;
- patients with primary and secondary malignancies of the liver, bile duct, gallbladder, pancreas, or periampullary malignant tumors;
- age ≥ 18 years;
- patients who underwent their first ERCP with endobiliary metal stent placement.

The exclusion criteria were as follows:

- patients with a resectable stage of disease;
- patients with a history of previous cholecystectomy;
- patients who had < 30 days of follow-up owing to transfer to other hospital or other causes;
- patients with failed cannulation or no stent placement.

**Fig. 1:**
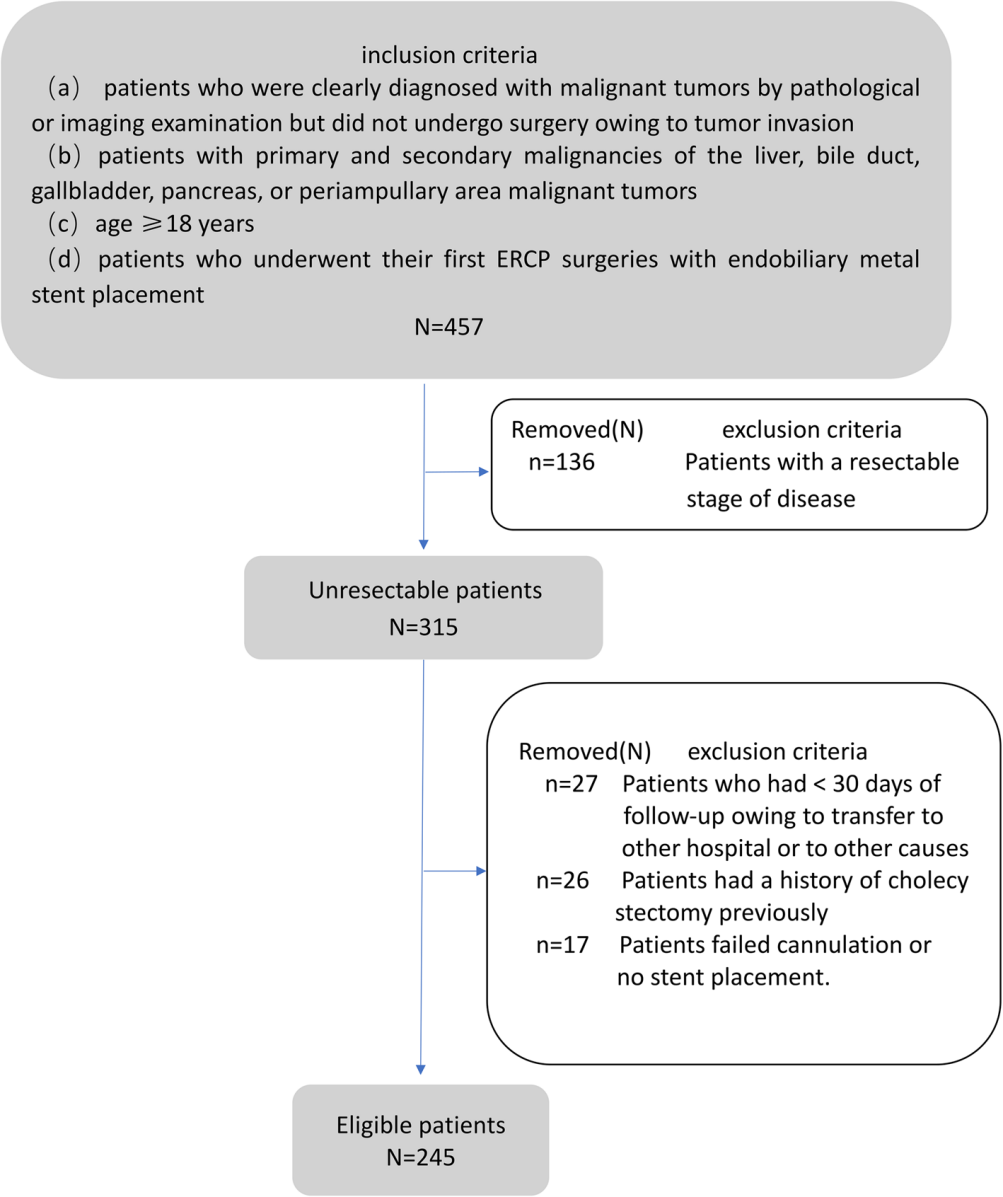
Detailed outline of patient screening process based on inclusion and exclusion criteria

All pertinent clinical and laboratory data before and after the ERCP were gathered. Additionally, the cross-sectional images and cholangiographic findings were reviewed. In cases where histological analyses were not possible, the diagnosis of MBO was based on a combination of imaging and clinical follow-up or histopathology alone. Patient selection flow is presented in Fig. 1.

### Endoscopic procedures for ERCP

Each patient underwent imaging, including computed tomography and magnetic resonance imaging, before biliary stent placement to plan effective drainage. All ERCPs were performed by doctors who had performed at least 200 ERCP procedures per year. Patients fasted for 8 h before surgery. General anesthesia was administered without tracheal intubation. Duodenoscopy was performed through the esophagus, stomach, and descending duodenum. A catheter was inserted at the duodenal papilla opening. Cholangiography was performed by attempting to aspirate bile, followed by a slow injection of iohexol. The level and length of the obstruction were clarified. Endoscopic sphincterotomy or papillary balloon dilation before biliary stent placement was allowed at the discretion of the endoscopist. ERCP was predominantly performed under sedation with intravenous midazolam or propofol in combination with meperidine. After successful stent placement, bile was drained into the duodenum. Postoperative abdominal radiography was performed to observe stent position and patency. Prophylactic antibiotics were routinely administered before ERCP.

The patients fasted from food and water for 24 h after surgery. The abdominal symptoms and signs were observed closely. Blood amylase level was rechecked on postoperative day 1, and liver function was checked at least once on postoperative days 1–7 to confirm the resolution of jaundice.

### Definitions

“High obstruction” refers to an obstruction between the common hepatic duct to the intrahepatic bile ducts; "Low obstruction" refers to an obstruction occurring in the common bile duct and far from the port hepatic. The 30-day mortality was defined as death within 30 days of ERCP. Successful drainage was defined as a decrease in TB of > 30% within 1 week after surgery or a total decrease in bilirubin in the later period close to the normal level (TB ≤ 17.1 μmol/L) [22]. Postoperative complications were defined as any procedure-related adverse event that occurred within 2 weeks, including bleeding, cholangitis, hemorrhagic complications, pancreatitis, and gut or bile duct perforations. Overall survival was calculated from the day of the procedure until either the day of death or the last follow-up day.

### Statistical methods

Our structured database contained 23 clinical variables. For data collection and processing, after completing statistical analysis and addressing missing data and noise, all predictive variables were standardized, Categorical variables are presented as percentages and numbers, while continuous variables are presented as mean ± standard deviation for normally distributed data and interquartile range (IQR) for skewed data. The chi-square test or Fisher’s exact test were used for comparing categorical variables, while the independent t-test or Mann–Whitney U test were used for comparing continuous variables. A *p*-value < 0.05 was deemed significant. Univariable logistic regression was utilized to identify clinical variables that impacted the study outcomes, which were then compared between patients who survived and those who did not. Odds ratios (OR) and 95% confidence intervals (CIs) were calculated for each variable. Variables that were found to be significant in the univariable analysis were included in the multivariable logistic regression. Significant variables in the multiple logistic regression were considered independent risk factors potentially affecting the outcome.

To avoid model overfitting or underfitting, LASSO regression was used to filter the features. The variance inflation factor (VIF) was assessed among the filtered variables. Variables with VIF > 5.0 were interpreted as multicollinear and not included in the final model analysis.

The final logistic regression model was developed based on the results of the LASSO regression and VIF. To improve the clinical utility of the model, a nomogram was developed where the coefficient of each variable in the regression model was used to assign points for each variable on the nomogram. Points were assigned to each variable by dividing its coefficient by the smallest coefficient and then rounding to the nearest integer. The total points for each patient were calculated by adding up the points assigned to each variable for that patient. Finally, the probability of an event occurring was estimated by calculating the distance from the total points to the probability axis.

We utilized 10-fold cross-validation and plotted the receiver operating characteristic (ROC) curve to assess the model’s discriminative ability and stability in the training cohort. Furthermore, the ROC curve was drawn for the test cohort to further assess the model’s performance. The area under the curve (AUC) values can range from 0.5 to 1.0, with 0.5 representing random chance and 1.0 indicating a perfect fit. 1000 bootstrap resampling iterations were employed separately in the training and testing cohorts to generate the calibration curve for the model. The calibration curve was then used to evaluate the model’s calibration performance. Calibration curves are a useful tool for validating and optimizing the performance of classification models. These curves typically have two axes: the x-axis represents predicted probabilities and the y-axis represents observed probabilities. If the model’s predictions match the actual observations, then the calibration curve should be close to the 45-degree diagonal line. If the curve is far from the 45-degree diagonal line, it indicates that the model has a bias. Additionally, decision curve analysis (DCA) is employed to assess the clinical utility of nomogram.

Considering the incidence rate of 30-day mortality events in patients, we classified patients into low- (below the 75th percentile), medium- (between the 75th and 90th percentiles), and high- (above the 90th percentile) risk groups based on their nomogram score percentile ranks. A Cox proportional hazards model was utilized to determine the hazard ratio and the probability of mortality based on the nomogram score groups. Cumulative survival rates over time were estimated using the Kaplan–Meier method. Statistical significance was set at a *p*-value < 0.05. All statistical analyses were performed using R version 4.21.

## Results

### Baseline characteristics

Twenty-three of 245 patients survived for less than 30 days. The baseline characteristics between the groups are presented in Table 1. The study cohort comprised 245 patients, who were randomly divided into training (*n* = 204) and test (*n* = 41) cohorts (Table 2).

**Table 1:** Baseline characteristics comparison between groups

**Variables**	**Total (*n* = 245)**	**Survived (*n* = 222)**	**Deceased (*n* = 23)**	***p***
**Sex, n (%)**				0.16
** Male**	103 (42.041)	97 (43.694)	6 (26.087)	
** Female**	142 (57.959)	125 (56.306)	17 (73.913)	
**Age, Median (Q1,Q3**^**a**^**)**	74 (65,80)	74 (64.25,80.00)	76 (65.5,80.5)	0.85
**Obstruction site, n (%)**				0.05
** Low obstruction**	198 (80.816)	183 (82.432)	15 (65.217)	
** High obstruction**	47 (19.184)	39 (17.568)	8 (34.783)	
**Stent placement, n (%)**				1
** One-stent placement**	233 (95.102)	211 (95.045)	22 (95.652)	
** Two-stent placements**	12 (4.898)	11 (4.955)	1 (4.348)	
**Lymph node metastasis, n (%)**				0.02
** No**	135 (55.102)	128 (57.658)	7 (30.435)	
** Yes**	110 (44.898)	94 (42.342)	16 (69.565)	
**Distant metastasis, n (%)**				< 0.01
** No**	158 (64.490)	151 (68.018)	7 (30.435)	
** Yes**	87 (35.510)	71 (31.982)	16 (69.565)	
**Cholangitis before stent insertion, n (%)**				1
** No**	200 (81.633)	181 (81.532)	19 (82.609)	
** Yes**	45 (18.367)	41 (18.468)	4 (17.391)	
**Post-ERCP complications, n (%)**				< 0.01
** No**	177 (72.245)	168 (75.676)	9 (39.130)	
** Yes**	68 (27.755)	54 (24.324)	14 (60.870)	
**Child–Pugh class, n (%)**				0.04
** A or B**	223 (91.020)	205 (92.342)	18 (78.261)	
** C**	22 (8.980)	17 (7.658)	5 (21.739)	
**Type of malignancy, n (%)**				0.12
** Gallbladder cancer**	119 (48.571)	111 (50.000)	8 (34.783)	
** Cholangiocarcinoma**	24 (9.796)	21 (9.459)	3 (13.043)	
** Ampullary cancer**	63 (25.714)	56 (25.225)	7 (30.435)	
** Other**	24 (9.796)	23 (10.360)	1 (4.348)	
** Pancreatic cancer**	15 (6.122)	11 (4.955)	4 (17.391)	
**Ascites, n (%)**				0.04
** No**	168 (68.571)	157 (70.721)	11 (47.826)	
** Yes**	77 (31.429)	65 (29.279)	12 (52.174)	
**White blood cell count (10**^**9**^**/L), Median (Q1,Q3)**	6 (4.5,7.5)	5.93 (4.46,7.27)	7.6 (5.46,9.16)	< 0.01
**Red blood cell count (10**^**12**^**/L), Mean ± SD**	3.584 ± 0.605	3.604 ± 0.599	3.384 ± 0.642	0.13
**Hemoglobin (g/L), Mean ± SD**	110.429 ± 17.702	111.05 ± 17.361	104.435 ± 20.149	0.14
**Platelet (10**^**9**^**/L), Median (Q1,Q3)**	187 (138,247)	187.5 (139,248)	158 (125.5,195.5)	0.16
**TB (mg/dL), Median (Q1,Q3)**	201.4 (113.0,288.2)	185.55 (108.90,276.32)	307.5 (239.40,381.05)	< 0.01
**ALT (u/L), Median (Q1,Q3)**	85 (46,149)	87.5(48.25,153.50)	74 (34.5,109.0)	0.07
**AST (u/L), Median (Q1,Q3)**	89 (52,145)	88.5 (53.00,144.75)	90 (52,149)	0.99
**Albumin (g/L), Mean ± SD**	34.59 ± 5.242	34.904 ± 5.16	31.557 ± 5.165	< 0.01
**Creatinine (μmoI/L), Median (Q1,Q3)**	64.8 (55.4,78.6)	64.1(55.25,76.58)	69.5 (61.55,89.00)	0.1
**PT (s), Median (Q1,Q3)**	13.8 (13.0,14.7)	13.7 (12.83,14.50)	14.4(13.85,16.35)	< 0.01
**Successful drainage**^**b**^**, n (%)**				< 0.01
** No**	114 (46.531)	94 (42.342)	20 (86.957)	
** Yes**	131 (53.469)	128 (57.658)	3 (13.043)	

*ERCP* Endoscopic retrograde cholangiopancreatography, *TB* Total bilirubin, *ALT* Alanine aminotransferase, *AST* Aspartate aminotransferase, *PT* Prothrombin time

Statistically significant with *p* < 0.05

^a^Interquartile range

^b^Defined by a decrease in TB > 30% within 1 week after surgery or a decrease in TB close to the normal level (TB $\le$ 17.1umol/l) in the later period

**Table2:** Patient characteristics and endoscopic interventions in the training and validation cohorts

**Variables**	**Total (*n* = 245)**	**Training cohort (*n* = 204)**	**Test cohort (*n* = 41)**
**Sex, n (%)**			
** Male**	103 (42.041)	82 (40.196)	21 (51.22)
** Female**	142 (57.959)	122 (59.804)	20 (48.78)
**Age, Median (Q1,Q3**^**a**^**)**	74 (65,80)	74 (64.75,80.00)	75 (65,79)
**Obstruction site, n (%)**			
** Low obstruction**	198 (80.816)	162 (79.412)	36 (87.805)
** High obstruction**	47 (19.184)	42 (20.588)	5 (12.195)
**Stent placement, n (%)**			
** One-stent placement**	233 (95.102)	193 (94.608)	40 (97.561)
** Two-stent placements**	12 (4.898)	11 (5.392)	1 (2.439)
**Lymph node metastasis, n (%)**			
** No**	135 (55.102)	110 (53.922)	25 (60.976)
** Yes**	110 (44.898)	94 (46.078)	16 (39.024)
**Distant metastasis, n (%)**			
** No**	158 (64.49)	131 (64.216)	27 (65.854)
** Yes**	87 (35.51)	73 (35.784)	14 (34.146)
**Cholangitis before stent insertion, n (%)**			
** No**	200 (81.633)	167 (81.863)	33 (80.488)
** Yes**	45 (18.367)	37 (18.137)	8 (19.512)
**Post-ERCP complications, n (%)**			
** No**	177 (72.245)	145 (71.078)	32 (78.049)
** Yes**	68 (27.755)	59 (28.922)	9 (21.951)
**Child–Pugh class, n (%)**			
** A or B**	223 (91.02)	186 (91.176)	37 (90.244)
** C**	22 (8.98)	18 (8.824)	4 (9.756)
**Type of malignancy, n (%)**			
** Gallbladder cancer**	24 (9.796)	93 (45.588)	26 (63.415)
** Cholangiocarcinoma**	63 (25.714)	24 (11.765)	0 (0)
** Ampullary cancer**	24 (9.796)	56 (27.451)	7 (17.073)
** Other**	15 (6.122)	19 (9.314)	5 (12.195)
** Pancreatic cancer**	119 (48.571)	12 (5.882)	3 (7.317)
**Ascites, n (%)**			
** No**	168 (68.571)	140 (68.627)	28 (68.293)
** Yes**	77 (31.429)	64 (31.373)	13 (31.707)
**White blood cell count (10**^**9**^**/L), Median (Q1,Q3)**	6 (4.5,7.5)	6.02 (4.60,7.54)	5.8 (4.2,7.3)
**Red blood cell count (10**^**12**^**/L), Mean ± SD**	3.584 ± 0.605	3.617 ± 0.578	3.416 ± 0.711
**Hemoglobin (g/L), Mean ± SD**	110.429 ± 17.702	111.309 ± 17.167	106.049 ± 19.803
**Platelet (10**^**9**^**/L), Median (Q1,Q3)**	187 (138,247)	183.5 (138.75,246.00)	207 (125,256)
**TB (mg/dL), Median (Q1,Q3)**	201.4(113.0,288.2)	198.55(115.40,288.05)	217.9(109.4,294.5)
**ALT (u/L), Median (Q1,Q3)**	85 (46,149)	82 (46.75,146.25)	97 (46,202)
**AST (u/L), Median (Q1,Q3)**	89 (52,145)	87.5 (52.00,141.75)	112 (64,146)
**Albumin (g/L), Mean ± SD**	34.59 ± 5.242	34.583 ± 5.226	34.622 ± 5.386
**Creatinine (μmoI/L), Median (Q1,Q3)**	64.8 (55.40,78.60)	64.65 (55.40,77.85)	66.3 (54.4,78.6)
**PT (s), Median (Q1,Q3)**	13.8 (13.0,14.7)	13.8 (12.9,14.7)	13.8 (13.1,14.9)
**Successful drainage**^**b**^**, n (%)**			
** No**	114 (46.531)	99 (48.529)	15 (36.585)
** Yes**	131 (53.469)	105 (51.471)	26 (63.415)
**30-day mortality**^**c**^**, n (%)**			
** No**	222 (90.612)	185 (90.686)	37 (90.244)
** Yes**	23 (9.388)	19 (9.314)	4 (9.756)

*ERCP* Endoscopic retrograde cholangiopancreatography, *TB* Total bilirubin, *ALT* alanine aminotransferase, *AST* Aspartate aminotransferase, *PT* Prothrombin time

Statistically significant with *p* < 0.05

^a^Interquartile range

^b^Defined by a decrease in TB of > 30% within 1 week after surgery or a decrease in TB close to the normal level (TB $\le$ 17.1umol/l) in the later period

^c^Defined by death within 30 days of ERCP

### Predictors for 30-day mortality

In the univariable analysis (Table 3), the following were identified as significant variables associated with 30-day mortality: lymph node metastasis (*p* = 0.016), distant metastasis (*p* = 0.00088), postoperative complications (*p* = 0.00053), Child–Pugh class C (*p* = 0.032), type of malignancy(*p* = 0.019), ascites (*p* = 0.029), TB (*p* = 0.00023), albumin (*p* = 0.0044), and PT (*p* = 0.044), On the other hand, the ALT levels (*p* = 0.049), successful drainage (*p* = 5e-04), and albumin levels (*p* = 0.0044) were found to have a negative correlation with 30-day mortality.

**Table 3:** Univariate and multivariate analyses of baseline variables for 30-day mortality prediction in the training cohort

**Variants**	**Univariate analyses**		**Multivariate analyses**	
	**OR (95%CI)**	***p***	**OR (95%CI)**	***p***
**Male**	2.2 (0.84–5.80)	0.11		
**Age**	1 (0.97–1.10)	0.72		
**High obstruction**	2.5 (0.99–6.30)	0.052		
**Two-stent placements**	0.87 (0.11–7.10)	0.9		
**Lymph node metastasis**	3.1 (1.2–7.9)	0.016	2.29 (0.59–8,98)	0.233
**Distant metastasis**	4.9 (1.9–12.0)	< 0.001	7.96 (1.44–44.10)	0.018
**Cholangitis before stent insertion**	0.93 (0.3–2.9)	0.9		
**Post-ERCP complications**	4.8 (2–12)	< 0.001	6.03 (1.52–24.01)	0.011
**Child–Pugh class C**	3.3 (1.1–10.0)	0.032	1.14 (0.16–8.11)	0.894
**Pancreatic Cancer**	1			
**Gallbladder cancer**	2 (0.49–8.10)	0.33	0.33 (0.03–3.40)	0.349
**Cholangiocarcinoma**	1.7 (0.6–5.0)	0.31	0.60 (0.13–2.71)	0.504
**Ampullary cancer**	0.6 (0.072–5.100)	0.64	1.06 (0.09–12.88)	0.966
**Other**	5 (1.3–19.0)	0.019	0.79 (0.10–5.99)	0.822
**Ascites**	2.6 (1.1–6.3)	0.029	1.25 (0.34–4.55)	0.738
**White blood cell count (10**^**9**^**/L)**	1.1 (1.0–1.2)	0.027	1.04 (0.93–1.16)	0.529
**Red blood cell count (10**^**12**^**/L)**	0.55 (0.27–1.10)	0.099		
**Hemoglobin (g/L)**	0.98 (0.95–1.00)	0.089		
**Platelet (10**^**9**^**/L)**	1 (0.99–1.00)	0.35		
**TB (mg/dl)**	1 (1–1)	< 0.001	1.01 (1.00–1.01)	0.009
**ALT (u/L)**	0.99 (0.98–1.00)	0.049	1.00 (0.99–1.01)	0.661
**AST (u/L)**	1 (0.99–1.00)	0.87		
**Albumin (g/L)**	0.88 (0.81–0.96)	0.004	0.90 (0.78–1.03)	0.131
**Creatinine (μmoI/L)**	1 (1–1)	0.15		
**PT (s)**	1.2 (1.0–1.4)	0.044	1.04 (0.87–1.24)	0.676
**Successful drainage**^**a**^	0.11 (0.032–0.380)	< 0.001	0.12 (0.03–0.54)	0.005

*ERCP* Endoscopic retrograde cholangiopancreatography, *TB* Total bilirubin, *ALT* alanine aminotransferase, *AST* Aspartate aminotransferase, *PT* Prothrombin time, *OR* Odds ratio, *CI* Confidence interval

Statistically significant with *p* < 0.05

^a^Defined by a decrease in TB of > 30% within 1 week after surgery or a decrease in TB close to the normal level (TB $\le$ 17.1umol/l) in the later period

In the multivariable analysis (Table 3), the following factors were identified as significant predictors of death within 30 days after ERCP: distant metastasis (OR 7.96; 95% CI, 1.44–44.10; *p* = 0.018), post-ERCP complications (OR 6.03; 95% CI, 1.52–24.01; *p* = 0.011), TB (OR 1.01; 95% CI, 1.00–1.01; *p* = 0.009) and successful drainage (OR 0.12; 95% CI, 0.03–0.54; *p* = 0.005).

**Fig. 2:**
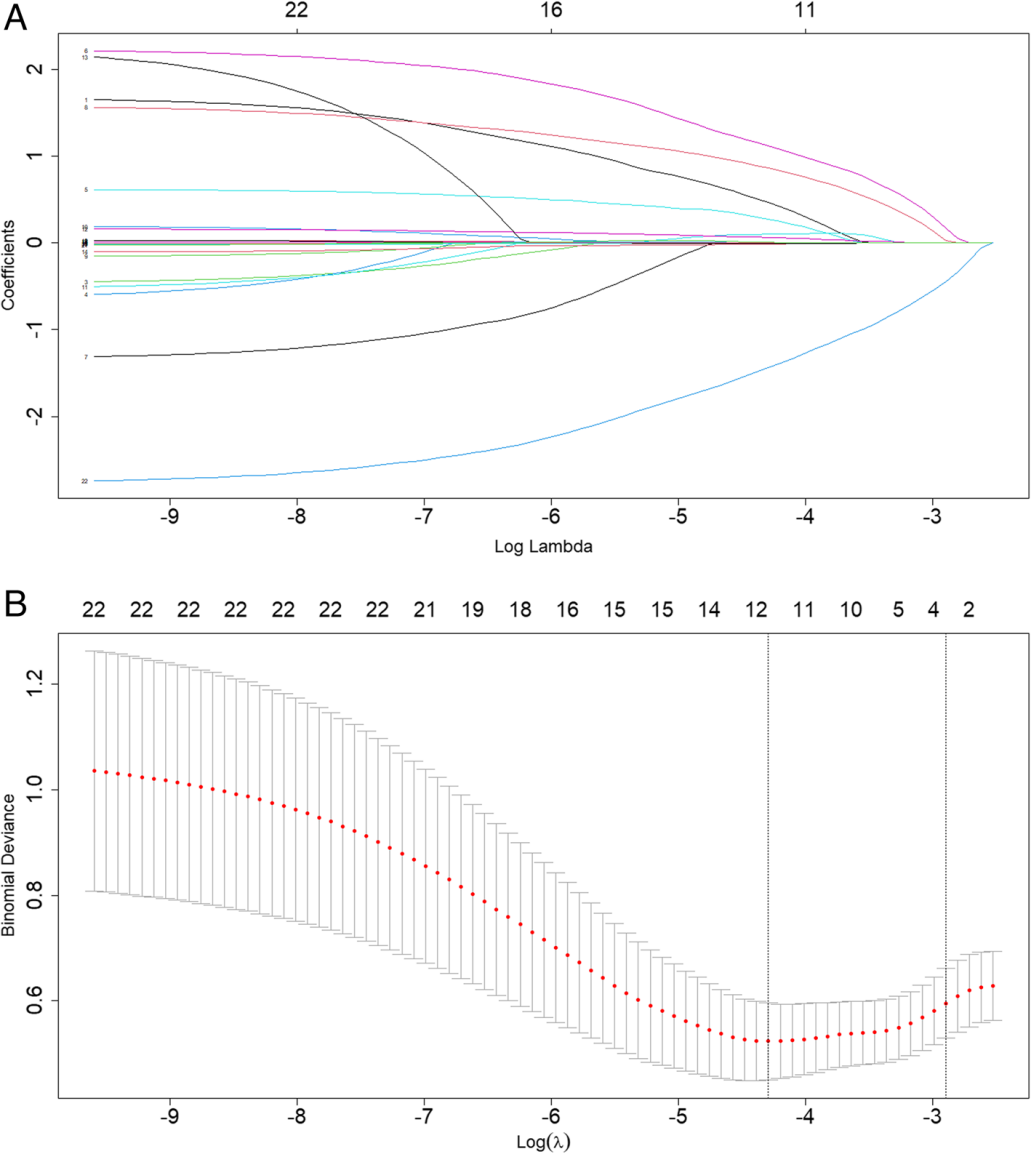
LASSO regression variable screening (**A**) The variation characteristics of the coefficient of variables; **B** the selection process of the optimum value of the parameter λ in the LASSO regression model by cross-validation

The parameters were screened using LASSO regression, and the coefficient variation characteristics of these variables are presented in Fig. 2A. To achieve a model with excellent performance and minimum variables, a 5-fold cross-validation method was utilized for iterative analysis. The obtained model had a λ of 0.05019702 (Fig. 2B). The results of lasso regression are consistent with the results of multivariable analysis, which strongly confirms the reliability of the variable selection outcome.

### Development of the 30-day mortality prediction nomogram

**Fig. 3:**
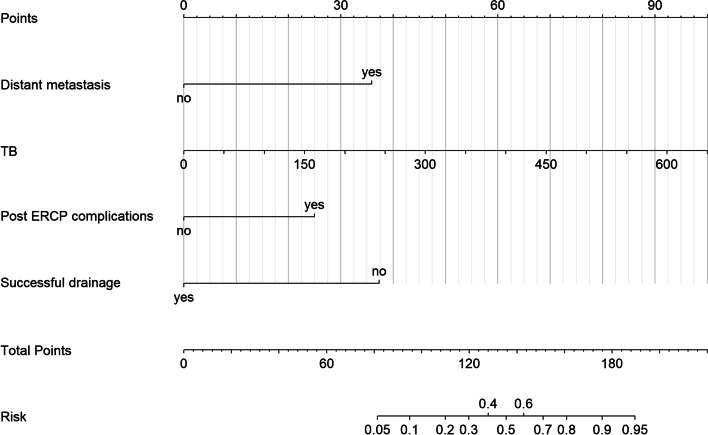
Nomogram used to predict short-term mortality after endoscopic endobiliary metal stent placement in patients with MBO. TB, total bilirubin. ERCP, endoscopic retrograde cholangiopancreatography. MBO, malignant biliary obstruction

A nomogram was developed based on the LASSO regression. The VIF values were all < 5, indicating no collinearity between the screened variables. Figure 3 shows the nomogram, which was generated using a logistic regression model that included all significant independent prognostic factors for 30-day mortality in the training dataset.

### Evaluation and validation of the nomogram

**Fig. 4:**
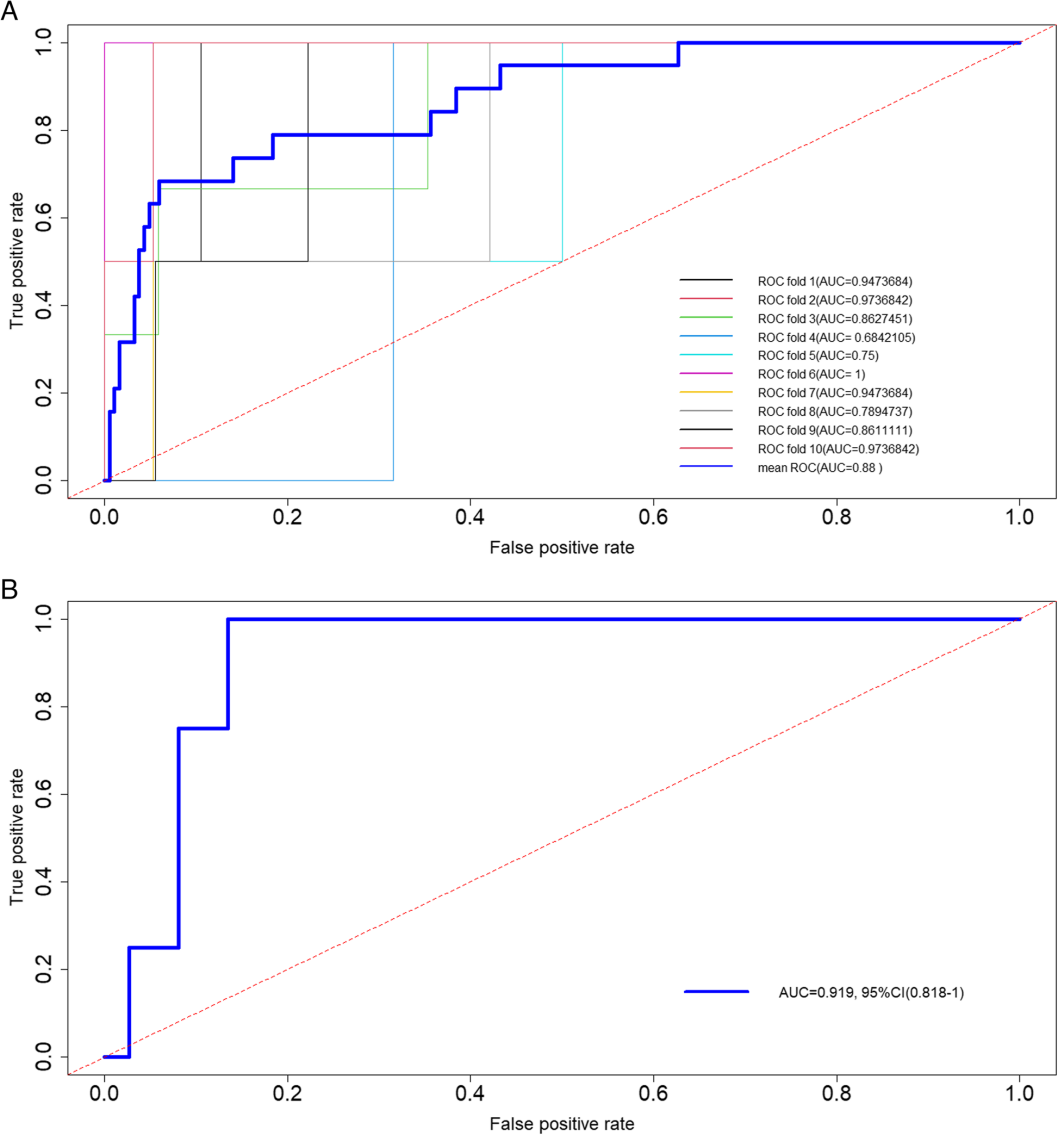
Area under the curve of the risk score. **A** The results of 10-fold cross-validation in the training cohort; blue lines represent the average ROC curve over the 10 folds. **B** The 10-fold cross-validation results for the test cohort. CI, confidence interval. ROC, receiver operating characteristic

We evaluated the model using a 10-fold cross-validation and obtained the following results: the model’s average AUC was 0.88 (standard deviation: 0.11). The ROC curves for 10 folds and the average ROC curve were plotted (Fig. 4A). These results indicate that the model has high accuracy and robustness in predicting the 30-day mortality of the patients, while maintaining a stable performance on different datasets. The ROC curve of the test set (Fig. 4B) had a AUC of 0.919 (95% CI: 0.818–1.000), further demonstrating the reliability of the results.

**Fig. 5:**
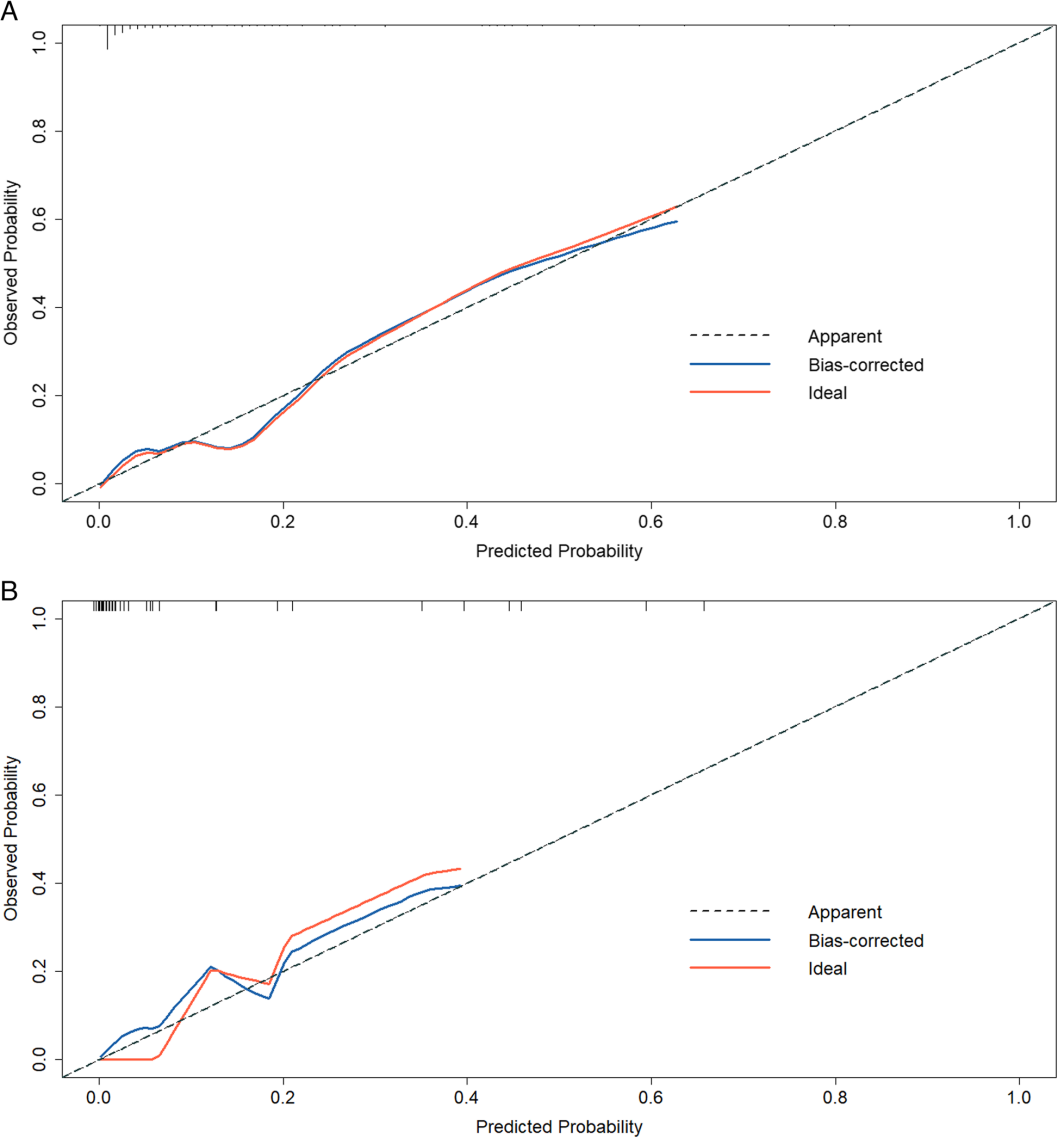
The calibration curve for nomogram. The nomogram-predicted short-term mortality probability is plotted on the x-axis, and the observed short-term mortality on the y-axis. A perfect prediction would correspond to a 45° grey dashed line. The blue solid line represents the cohort (training or test), and the red solid line represents the bias corrected by bootstrapping (B = 1000 repetitions), indicating the observed nomogram performance. Calibration curves for predicting short-term mortality in the training (**A**) and test (**B**) cohorts. MBO, malignant biliary obstruction

After generating the calibration curve using 1000 iterations of Bootstrap resampling for both the training and test datasets (Fig. 5), we evaluated the calibration performance of the model using the calibration curve. Based on our evaluation, the model demonstrated good calibration performance.

**Fig. 6:**
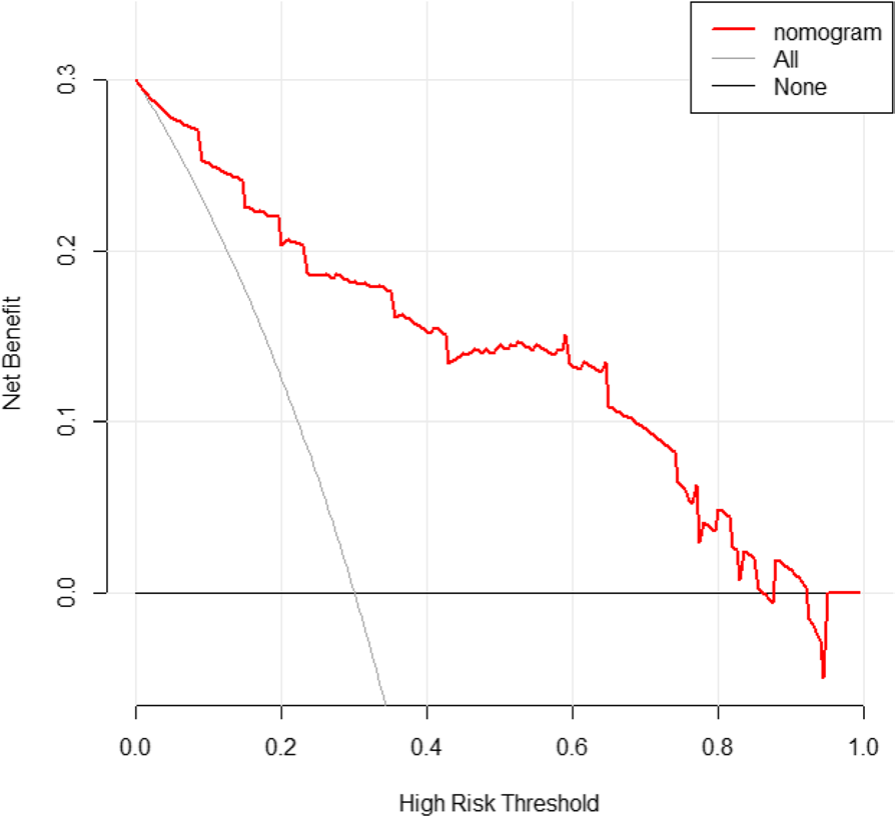
DCA of the nomogram. DCA, decision curve analysis

DCA is utilized to evaluate the clinical utility of the model (Fig. 6). It demonstrates that the nomogram model exhibits higher net benefits across a wide range of threshold probabilities compared to both full treatment and no treatment scenarios.

### Risk stratification based on the nomogram

**Fig. 7:**
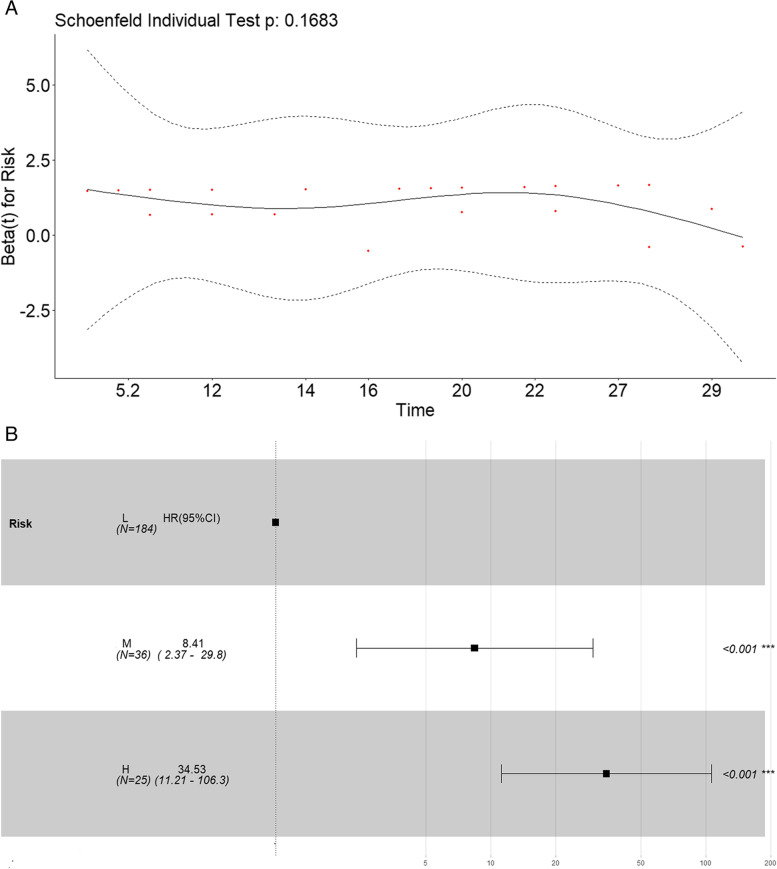
Schoenfeld residuals analysis of risk groups. **A** Hazard ratio in different risk groups. **B** L, low-risk group. M, moderate-risk group. H high-risk group.95% CI, 95% confidence interval

Finally, we used the nomogram to perform risk stratification. Patients with probability total scores of < 90, 90–120, and $\ge$ 120 were assigned to the low-, moderate-, and high-risk groups, respectively. The proportionality hazard assumption was assessed using Schoenfeld residual analysis. In Fig. 7, the *p*-values for the risk groups were presented (Fig. 7A). The results indicated that the risk groups met the proportionality hazard assumption. Compared to the low-risk group, the middle-risk group and high-risk group had HRs of 8.41 (95% CI, 2.37–29.8; *p* < 0.001) and 34.53 (95% CI, 11.21–106.3; *p* < 0.001), respectively (Fig. 7B).

**Fig. 8:**
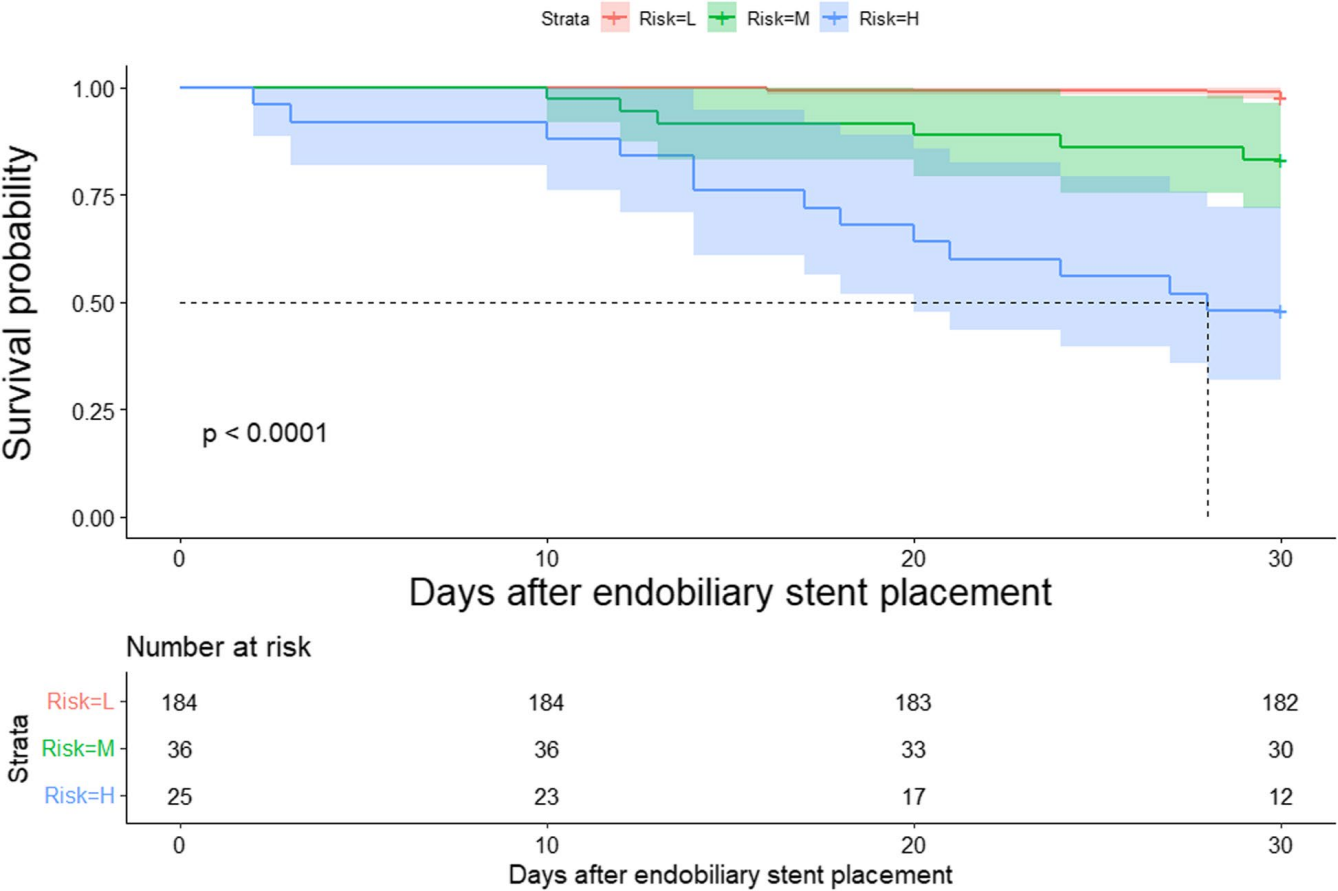
Kaplan–Meier survival curves for nomogram-grouped patients with MBO after endoscopic endobiliary metal stent placement. The *p*-value (< 0.001) was determined using the log-rank test. L, low-risk group. M, moderate-risk group. H high-risk group. MBO, malignant biliary obstruction

The Kaplan–Meier survival curves separated by nomogram-based grouping are depicted in Fig. 8, revealing a significantly lower 30-day mortality in the low-risk group compared to that of the high-risk group (*p* < 0.001).

## Discussion

Our study demonstrates that post-ERCP complications, distant metastasis, TB, and successful drainage are significant independent predictors of 30-day post-ERCP mortality in patients with MBO. We should pay attention to and reasonably dispose of these independent factors to improve surgical outcomes and reduce the financial burden on the patients.

Post-ERCP complications are adverse events following surgery for MBO and may affect the short-term mortality rates of patients. Previous studies have shown that post-ERCP complications are significantly associated with short-term mortality in these patients. For instance, a retrospective study found that patients with postoperative cholangitis had significantly higher early mortality rates (6.5% vs. 0.5%, *p* < 0.001) [23]. Another study suggested that post-ERCP complications are one of the independent risk factors for short-term mortality in patients with MBO [24]. Several factors may explain why post-ERCP complications can affect short-term mortality in these patients. First, these complications can prolong hospitalization, increasing the patients burden and pain [25, 26]. Second, post-ERCP complications can weaken patients’ physical condition, increasing the risk of short-term death [27, 28]. In conclusion, post-ERCP complications are closely related to short-term mortality in patients with MBO. Therefore, active measures should be taken to prevent and manage such complications during treatment, in order to reduce the risk of short-term mortality in these patients.

Poor tumor staging is considered an unfavorable factor affecting the survival of patients with MBO. Due to difficulties in obtaining tumor samples and low diagnostic sensitivity, most studies [29, 30] use the Bismuth–Corlette classification and metastatic status to determine tumor stages. Metastatic and advanced tumors are more likely to result in shorter survival times in patients with MBO [31]. Liver metastases are an independent factor influencing the 12-month survival of patients with unresectable hilar cholangiocarcinoma (HCCA) [32]. Tumor stage is an independent risk factor for survival in patients with HCCA [33]. The results of the multivariable analysis in our study showed that distant metastasis was an independent adverse factor affecting survival, which is consistent with previous studies.

Furthermore, successful drainage was significantly associated with 30-day mortality. Adequate bile drainage is considered a technical success for biliary stenting and is associated with increased survival rates [22, 32–37]. However, the number of stents was not associated with 30-day mortality. Several studies have reported similar effects of unilateral and bilateral stents on bile drainage [38–40]. In a small retrospective study of 46 consecutive patients who underwent palliative endoscopic biliary stenting for MBO, the overall stent patency rate was significantly higher in the group that received bilateral stents than that of the unilateral stenting group [41]. This may be because the unilateral stent does not provide adequate drainage from the obstruction site. Stenting is performed to provide adequate drainage, and, regardless of the number of stents implanted, the final bile connection status is always the same as long as adequate drainage is ensured. Therefore, bile drainage efficiency should not be influenced by the number of stents used.

The nomogram was constructed with distant metastasis, TB, Post-ERCP complications, and successful drainage as relevant factors. Our study demonstrated that the prediction model performed well in both discriminating and calibrating the training cohort and was subsequently validated in the test cohort, showing consistent diagnostic performance and confirming its reliability. To enhance the clinical utility of the prediction model, we divided the predicted risk scores into three groups. Figure 8 depicts the reliability of this classification, which could aid physicians in deciding optimal palliative strategies for patients with unresectable MBO. For instance, patients with a total score > 120 have a high likelihood of death within 30 days of stent placement. In our scoring system, patients can reach this level even before surgery, indicating that their risk may have been underestimated. For such patients, surgery should be postponed unless it is certain that it will significantly improve the patient’s symptoms. Furthermore, a preoperative multi-disciplinary treatment plan should be devised to minimize the short-term risk of death by a combination of liver protection options. If invasive interventions are performed on such patients, plastic stents may be a better choice. Patients who reach this score after stent placement should remain in hospital observation time for an appropriately extended period to prevent adverse events. In contrast, patients with probability total scores < 90 had a low probability of death 30 days after stent placement. Biliary drainage can benefit these patients, and metal stents are preferred. For patients with a total probability score between 120 and 90, the treatment approach should be determined based on the patient’s performance status and the physician’s discretion.

This study has multiple strengths. One of the main advantages of this study was the inclusion of a substantial cohort of patients with unresectable MBO, as well as a lengthy period of data collection. Furthermore, the study is, to our knowledge, the first to use LASSO regression to screen features and develop a 30-day mortality prediction model, which was subsequently internally validated using a test cohort. Finally, in practice, information regarding the variables used in the nomogram can be easily obtained from patients with MBO. Using our predictive model, clinicians can immediately and accurately predict a prognosis and obtain useful information regarding postoperative treatment.

However, our study also has some limitations. Firstly, being a retrospective study, we might have missed some significant clinical features associated with mortality in our cohort. Secondly, the potential for unmeasured bias cannot be ruled out, and therefore, the prediction model needs to be externally validated in a larger prospective cohort to confirm the accuracy of the model in predicting 30-day mortality after stent placement.

## Conclusions

The prognosis for patients with MBO is poor. Biliary drainage can benefit these patients and, therefore, should be performed. The choice of treatment options for this group of patients should consider both cost-effectiveness and the expected length of survival. We identified four variables affecting the survival of patients with MBO and constructed a nomogram to assist clinical decisions. All the variables were objective and easily accessible. The nomogram can help identify which patients with MBO would benefit from ERCP in terms of survival. This allows us to better select future ERCP candidates and develop a reasonable treatment plan.

## Data Availability

The data that support the findings of this study are available from the corresponding author, Huangbao Li, upon reasonable request.

## Abbreviations

MBO: Malignant biliary obstruction
ERCP: Endoscopic retrograde cholangiopancreatography
PS: Plastic stents
MS: Metal stents
ML: Machine learning
PT: Prothrombin time
TB: Total bilirubin
ALT: Albumin, alanine aminotransferase
AST: Aspartate aminotransferase
OR: Odds ratios
CI: Confidence interval
VIF: Variance inflation factor
AUC: Area under the receiver operating characteristic curve
ROC: Receiver operating characteristic
HCCA: Hilar cholangiocarcinoma
DCA: Decision curve analysis
